# Occurrence of Fox Squirrels Influenced by Fine‐Scale Landscape Characteristics on a College Campus

**DOI:** 10.1002/ece3.70488

**Published:** 2024-10-27

**Authors:** Daniel A. Whitman, Ty J. Werdel

**Affiliations:** ^1^ Department of Biology Macalester College Saint Paul Minnesota USA; ^2^ Department of Rangeland, Wildlife, and Fisheries Management Texas A&M University College Station Texas USA

**Keywords:** fox squirrel, landcover, line transect, *Quercus*, synanthrope, urban

## Abstract

Urbanization and urban sprawl generally degrade and diminish wildlife habitat, threatening to extirpate local populations. However, certain synanthropic species (e.g., coyotes, white‐tailed deer, and squirrels) are able to persist in urban environments and may even occur at greater densities than they do in their natural habitats. Eastern fox squirrels (*Sciurus niger*) are large tree squirrels that are known to be present in greater densities within urban areas. To determine how landscape characteristics may affect fox squirrel presence, we conducted line‐transect surveys along sidewalks on the Texas A&M University—College Station campus to record presence of fox squirrels and nearest tree species. We calculated Jacobs’ index of selectivity (*D*) for use of trees by fox squirrels along the transects. Squirrel density was calculated for all transects and modeled using linear regression with environmental (e.g., tree density) and survey (e.g., transect distance) variables. Fox squirrels preferred only a small number of the available tree species, primarily *Quercus* and *Ulmus* species. Observed fox squirrel density significantly increased with time of day, temperature, density of oaks (*Quercus* spp.), and density of all trees, and decreased with distance and area of the transect. These results suggest that even when urban areas contain suitable habitat, use of urban environments by wildlife is still highly selective and dependent on specific habitat requirements.

## Graphical abstract

Synanthropic species are able to persist in urban environments and may even occur at greater densities than they do in their natural habitats. To determine how landscape characteristics may affect presence of synanthropic Eastern fox (*Sciurus niger*) squirrel presence, we conducted line‐transect surveys along sidewalks on the Texas A&M University—College Station campus to record presence of fox squirrels and nearest tree species. Fox squirrels preferred only a small number of the available tree species, primarily *Quercus* and *Ulmus* species, and observed fox squirrel density significantly increased with time of day, temperature, and tree and oak density, and decreased with length and area of the transect.

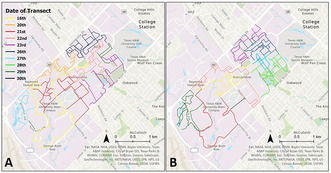

## 1 Introduction

Urbanization has drastically increased across the globe, expanding by 58,000 km^2^ from 1970 to 2000 and projected to spread over at least 430,000 km^2^ more by 2030 (Seto et al. 2011). Urban intensification has been shown to degrade habitat quality (Bai et al. 2019) and fragment remaining habitat (Gibb and Hochuli 2002; Liu, He, and Wu 2016; Xu et al. 2018), which can decrease genetic diversity by reducing population connectivity (Blanchet et al. 2010; McCleery 2010). In general, urbanization has been associated with declines in species diversity and relative abundance across diverse taxonomic groups (Baker et al. 2003), including arthropods (Gibb and Hochuli 2002), mammals (McKinney 2008), and birds (Xu et al. 2018), due to factors such as lower vegetative diversity, replacement of natural habitat by exotic plant species, and coverage by impervious surfaces (Green and Baker 2003; McKinney 2008). Even in urban habitat fragments with high woody cover and plant species heterogeneity, small mammal diversity may still be lower than comparable habitat outside of urban areas (Fernández and Simonetti 2013). Large carnivores rarely survive in urban areas (Crooks 2002; McCleery 2010), which can increase mesopredator populations and alter community composition (Chupp et al. 2013; Gibb and Hochuli 2002).

However, some species are not only able to persist in urban environments, but may even have greater population densities than they do in their natural habitat; these species are known as synanthropes (McCleery 2010). Urban synanthropes are often omnivores, early successional species, habitat generalists, edge specialists, or non‐native species (Johnston 2001; McCleery 2010). Adaptations to urban living may include longer breeding seasons, increased nocturnality, diet changes, and habituation to human presence (McCleery 2010; Riley et al. 2003; Ritzel and Gallo 2020; Santini et al. 2019). Multiple factors can drive greater population densities in synanthropes. Urban areas can provide a more abundant, stable, and nutrient‐rich diet than rural areas via anthropogenic food sources or decrease predation risk due to hunting restrictions or extirpation of natural predators (Bateman and Fleming 2012; Hansen et al. 2020; McCleery 2010; Shochat, Lerman, and Fernández‐Juricic 2010). Nevertheless, synanthropes may not use urban environments indiscriminately. Home range boundaries may follow those of remnant natural areas as individuals avoid areas of greatest human presence (Gehrt, Anchor, and White 2009) and wildlife persistence can vary between different types of urban landscapes (Gallo et al. 2017). Where synanthropic populations are high, they may cause human–wildlife conflicts by eating people's food, spreading diseases, attacking pets, or destroying property; however, the presence of urban wildlife reportedly increases people's sense of well‐being, and childhood exposure to nature can increase future support for wildlife conservation by fostering positive attitudes (Feinsinger 1987; Perry et al. 2020).

Eastern fox squirrels (*Sciurus niger*; hereafter, fox squirrels) are a species native to the eastern and midwestern United States with large urban populations that may be described as synanthropic (McCleery 2009a, 2009b, 2010). Fox squirrel populations have been found in greater densities in fragmented areas as individual home ranges shrink (Koprowski 2005). The endangered Big Cypress subspecies (*S. n. avicennia*) maintains denser populations on golf courses because of native species plantings (Jodice and Humphrey 1992). Urban populations face less predation than rural populations (McCleery 2010) and have higher juvenile survival rates (McCleery 2009a, 2009b) but do not differ in long‐term survival rates because they face higher anthropogenic mortality than rural populations (McCleery 2010). Furthermore, though generally solitary and territorial in native habitats (Moore 1957), urban fox squirrels tolerate each other, even in feeding areas, and may take advantage of group feeding as a predator defense (Jodice and Humphrey 1992).

While fox squirrels prefer foraging in relatively open areas over dense, closed‐canopy forests (Sexton 1990), density of fox squirrels depends on density of trees, and declines in preferred tree species can restrict population density (Moore 1957). Areas with dense undergrowth and few mast‐producing trees may exclude fox squirrels and limit urban population connectivity (Ditgen, Shepherd, and Humphrey 2007; Jodice and Humphrey 1992). Fox squirrels forage more effectively in areas with vegetative cover (Schmidt and Brown 1996), select food sources based on proximity to cover (Brown and Morgan 1995), and generally avoid completely exposed areas (Ditgen, Shepherd, and Humphrey 2007). Previous research in natural habitats has not found a high degree of selectivity for specific tree species within habitat patches (Perkins and Conner 2004; Brown and Batzli 1984). However, in urban environments, fox squirrels may be more selective by tree species in order to avoid dense understory or highly developed patches (Ditgen, Shepherd, and Humphrey 2007).

Previous research on fox squirrels on the College Station campus of Texas A&M University revealed that use of urban habitat by fox squirrels was positively influenced by the presence of mature oak trees, and fox squirrels were less active on paved areas, in favor of using grassy areas with greater canopy cover (McCleery 2010). For this study, we expanded on these findings by investigating whether squirrel density correlates not only with oak tree density but also with overall tree density. While oaks, especially *Quercus virginiana*, were expected to be a significant driver of habitat selection, we predicted that the density of all trees, including non‐oak species, would also positively influence squirrel density due to their role in providing cover, foraging opportunities, and suitable microhabitats. We further considered squirrels' microhabitat preferences by calculating Jacobs' index of selectivity (*D*) for all tree species used by squirrels. Additionally, we recognize the value in repeating similar experiments in the same system to assess whether results remain consistent over time and whether findings on the relationship between squirrel density and tree density, particularly oak density, is replicable.

## 2 Methods

### 2.1 Study Area

**FIGURE 1:**
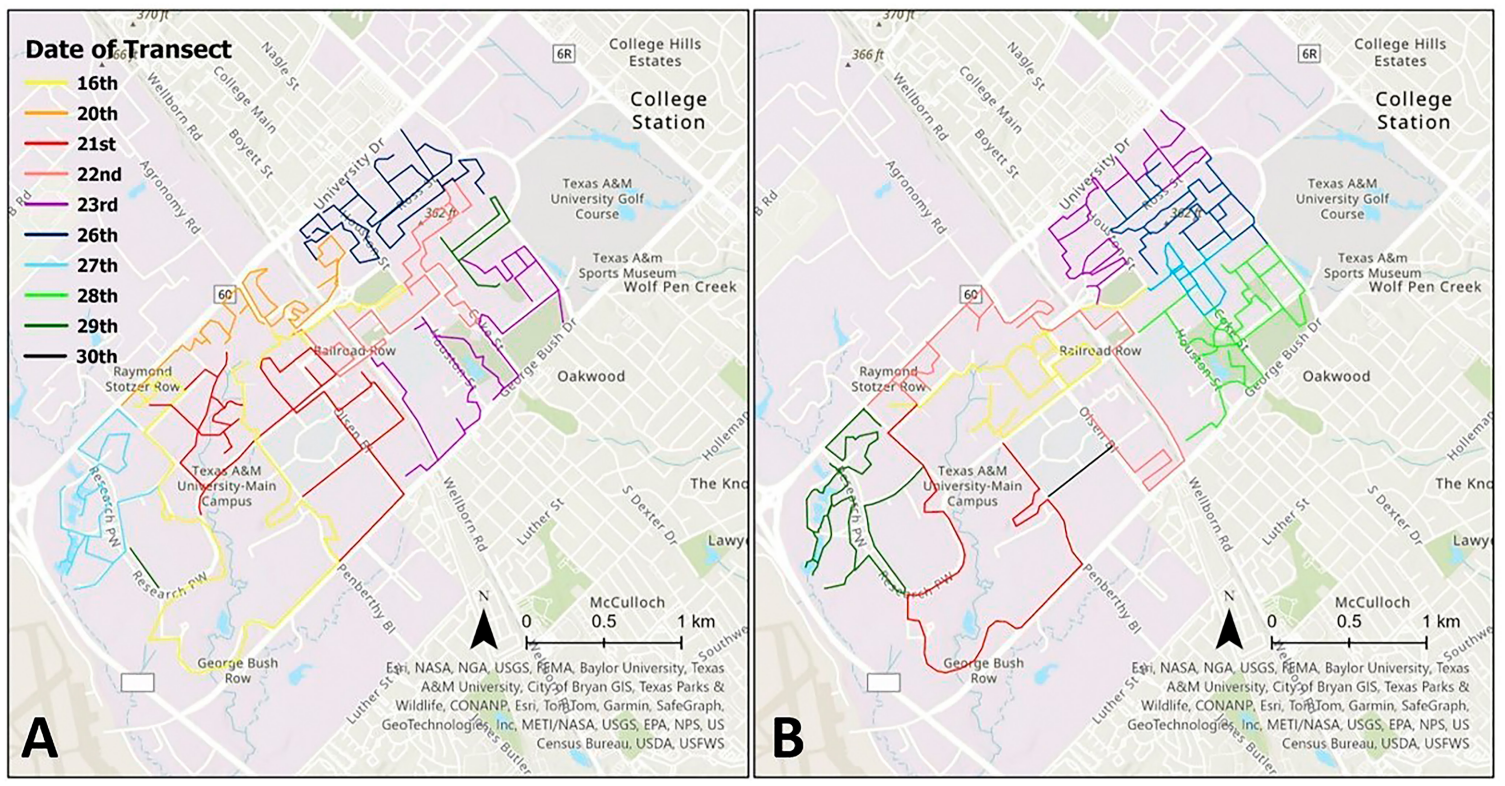
Study area at Texas A&M University's College Station campus, with lines showing survey routes in morning (A) and evening (B). Line colors represent different days. Morning and evening survey routes were walked one time each.

This study was conducted on Texas A&M University's College Station campus (580 ha) in Brazos County, Texas (Figure 1). Prior to European colonization, College Station was covered in Post Oak Savannah dominated by *Quercus stellata* (Campbell 1925; Srinath and Millington 2016). Since then, woody understory has encroached on the Post Oak Savannah, much of the savannah has been cleared, and bottomland tree species, including *Ilex opaca*, *Q. macrocarpa*, *Q. virginiana*, and *Ulmus crassifolia*, have been planted on the Texas A&M University campus (Campbell 1925; Srinath and Millington 2016). Landcover of the Texas A&M University campus is primarily composed of buildings and impervious surfaces, such as sidewalks and roads, interspersed with grassy lawns, gardens, tree plantings, and some remnant oak savannah and bottomland forest along streams.

From 1991 to 2020, average annual temperature in College Station was 69.4 °F, with an average of 82.6 °F in June, and average annual precipitation of 41.75 in., with an average of 4.01 in. in June (NOAA NCEI 2024).

Possible predator species in the area include Gray Fox (*Urocyon cinereoargenteus*), Coyote (*Canis latrans*), Red‐tailed Hawk (*Buteo jamaicensis*), Red‐shouldered Hawk (*Buteo lineatus*), owls (*Strigidae* spp.), feral or domestic cats (*Felis catus*), and humans (McCleery 2009a, 2009b; Moore 1957).

### 2.2 Surveys

During June 16 to June 30, 2024, squirrels were visually counted during walking surveys along established sidewalks and trails. All data were collected by a single observer. Binoculars (Nikon Monarch 5 8 × 42, Nikon, Inc., Melville, NY, USA) were used to search for and observe squirrels. Due to habituation, squirrels did not flush unless approached very closely, in which case, recorded location was the spot where the squirrel was first seen. Surveys were designed to cross through all areas of campus, covering as much area as possible that could be observed from the sidewalks and roads. The area along each sidewalk or road was surveyed twice: once in the morning between 7 a.m. and 12 p.m. (UTC‐6) and again in the evening between 5 p.m. and 8 p.m., though not necessarily on the same day. The daily route was selected at random. Surveys were timed to match estimated peak fox squirrel activity (Hicks 1949; Jodice and Humphrey 1992; Koprowski and Corse 2005; Moore 1957). Each squirrel sighting was marked to exact geographic location in a point layer in ArcGIS Field Maps (v. 23.2.2, Esri, Redlands, CA, USA), and date, time, species of nearest tree, if squirrel occupied a tree, and ambient temperature; (Apple Weather App; The Weather Channel/National Weather Service/NOAA); were recorded. If a squirrel switched behaviors or moved between two trees or between a tree and another substrate, this was noted as an additional behavioral observation. We recorded walked routes as a layer of line segments in ArcGIS Field Maps or recorded on a field data sheet and later transcribed in ArcGIS Pro (v. 2.8.0, Esri, Redlands, CA, USA).

### 2.3 Covariate Characterization

We divided line segments into transects (*n* = 75) based on the time of the squirrel sightings and average walking pace during each survey, since Field Maps did not collect exact time. Because evening transects were not originally plotted in Field Maps and had to be transcribed and assigned to date and time after the fact, timing and extent of the evening transects is potentially less accurate than for morning transects. Each of the transects was continuous and represented the total time walked within a half hour time frame, with the exception of two distant, noncontinuous transects within the same half hour and one noncontinuous transect. In both cases, only some of the time during the half hour was spent looking for squirrels. Line segments sketched in ArcGIS Field Maps were adjusted to make sure they lay across sidewalks and roads and did not cross over buildings. Doubling back was also removed from all transects, so that transects represented one‐way distance only. Categorical time of day was defined for all transects as early morning (7:00–9:30 a.m.), late morning (9:30 a.m. to 12:00 p.m.), early evening (5:00–6:30 p.m.), or late evening (6:30–8:00 p.m.). These periods were chosen to capture the likely bimodal activity pattern of fox squirrels, with peaks in activity during cooler parts of the day. Previous studies have documented that fox squirrels are most active in the early morning and late afternoon, with reduced activity during the midday heat, particularly in warmer climates like Texas (Hicks 1949; Jodice and Humphrey 1992). We avoided surveying during midday (12:00–5:00 p.m.) when temperatures often exceed 35°C (95 °F), which likely suppresses squirrel activity. Temperatures were converted from Fahrenheit to Celsius using the Convert function in Excel.

Transects and sightings were separated into morning and evening layers using the Feature Class to Feature Class tool (ArcGIS Pro). In order to reflect different visibility along roads and sidewalks on campus, effective strip width was estimated for all transects using distance to farthest squirrel seen along the transect. If no squirrels were seen along the transect, we estimated visibility, generally by distance to nearest tree or nearest building or effective strip width on closest transect. We created buffer layers (“Buffer”; ArcGIS Pro) containing polygons representing the area surveyed along each transect, based on transect distance and effective strip width. We calculated area (geodesic) in square kilometers and distance (geodesic) in kilometers of the area within each effective strip width (“Calculate Geometry”; ArcGIS Pro). Temperature was only recorded when squirrels were seen. For transects with squirrel observations, the minimum and maximum recorded temperatures were specified. For transects without squirrel observations, this information was estimated based on temperature of transects before and after.

Layers containing campus‐wide tree data, including tree species, collected from 2006 to 2012 and in 2019, were combined into a single layer using the Merge tool (Unknown Author 2023; ArcGIS Pro). The Select Layer by Attribute tool was used to create a layer containing only oak trees (*Quercus* spp.; ArcGIS Pro). The Spatial Join tool was used to obtain the total number of trees and total number of oak trees within each transect buffer (ArcGIS Pro). Transect buffers were combined into a single layer using the Merge tool, and the Spatial Join tool (ArcGIS Pro) was used to find all the individual trees and all the individual oak trees within the observed area. The Spatial Join tool was also used to find the number of total trees and the number of oak trees within each transect buffer. Number of squirrels, trees, and oak trees along each transect were divided by the area within effective strip width to obtain density of squirrels, trees, and oaks in number per square kilometer. Proportion of oak trees was also calculated as number of oak trees divided by total number of trees. Because previous research found that urban fox squirrels selected for oak trees (McCleery et al. 2007), we calculated density and proportion of oak trees to consider the specific effect of oaks on observed squirrel densities.

The tree layers did not include some trees observed in the field that were closest to or occupied by squirrels. Merging the tree layers potentially created duplicates of some trees because the two tree survey layers may not be completely exclusive, although no overlapping points were seen in the tree layers.

### 2.4 Statistical Analysis

Jacobs' indices of selectivity (*D*) (Jacobs 1974) was used to calculate selection of individual trees by species and genera, for trees in the Aggie Tress Layer were only identified to genus and for genera with several species present. Jacobs' index has been used in studies of microhabitat selection (Kacoliris, Eleonora Celsi, and Laura Monserrat 2009) and is more sensitive than simple selection ratios (Kauhala and Auttila 2010). Jacobs' was calculated as *D* = (*r* − *p*)/(*r* + *p* − 2*rp*), where *r* equals proportion of trees of the given species that were used and *p* equals proportion of total trees within effective strip width of the transects that were the given species. Species and genera were considered preferred at *D* > 0 and avoided at *D* < 0. Selection indices were calculated for trees occupied by squirrels, as well as for nearest tree to squirrel to investigate selection for purposes like shade or temporary foraging cover and not necessarily as a food source or nest sight Trees occupied by a squirrel were also counted as nearest trees. If a tree was occupied or nearest to more than one squirrel, this was counted as more than one instance of selection.

Statistical analysis was completed using R statistical software (v. 4.3.2, R Core Team 2022), in RStudio (v. 2023.12.0 + 369). A one‐way ANOVA was used to test for bivariate relationship between squirrel density and categorical time of day (early and late morning and early and late evening), and a Tukey's HSD test was used to test for significant pairwise differences. We checked the diagnostics of our ANOVA using Shapiro–Wilk and Bartlett's tests. Because the Shapiro–Wilk test did not confirm normal distribution (*p* = 1.59^e‐13^), and the Bartlett's test did not confirm homogeneity of variances across groups (*p* = 6.2^e‐11^), we conducted a Kruskal–Wallis rank sum test. Pearson correlation coefficients (PCC) were calculated for all combinations of parameters before linear regression (Table 1). Parameters with correlation coefficient > 0.6 were not used in combination. Linear models of squirrel density were constructed using minimum and maximum temperature, density of all trees, density of oak trees, proportion of oaks, hour of day, distance and area of transect, and duration of survey effort, including interaction terms between parameters. Because previous research has shown bimodal peaks in daily fox squirrel activity (Hicks 1949), we also treated hour as a 2nd degree polynomial term to potentially fit this pattern. Null and global models were also constructed (Table 2). The most supported and parsimonious models were selected by Akaike information criterion (AICc; adjusted for small sample size) values. We checked selected models using normal Q–Q plots of the residuals.

**TABLE 1:** Pearson correlation coefficients (PCC) between all pairs of model parameters. Parameters with correlation coefficient > 0.6 were not used in combination during modeling.

Parameter 1	Parameter 2	PCC
Minimum temperature	Hour	0.9570
Maximum temperature	Hour	0.9609
Hour	Duration	−0.0251
Hour	Estimated minimum temperature	0.9538
Hour	Distance	0.2937
Hour	Area	0.3826
Estimated minimum temperature	Duration	−0.0963
Estimated minimum temperature	Distance	0.1638
Estimated minimum temperature	Area	0.2778
Tree density	Hour	0.0904
Tree density	Duration	0.0002
Tree density	Distance	−0.0834
Tree density	Area	−0.1239
Tree density	Oak density	0.8079
Tree density	Proportion of oaks	−0.0954
Oak density	Hour	0.0907
Oak density	Duration	−0.1133
Oak density	Distance	−0.1285
Oak density	Area	−0.2755
Oak density	Proportion of oaks	0.4197
Distance	Area	0.6549
Distance	Duration	0.5298
Area	Duration	0.3050
Proportion of oaks	Distance	−0.0756
Proportion of oaks	Area	−0.3008

**TABLE 2:** Summary of evaluated models. Models using area instead of distance, oak density instead of tree density, and minimum temperature or categorical time of day instead of hour are not shown.

Hour + distance
Tree density + hour + distance
Tree density + hour + oak proportion
Oak proportion × oak density
Oak proportion × tree density + distance
Oak proportion × oak density + hour + distance
Tree density × hour
Tree density × hour + distance
Tree density × hour + duration
Tree density + (hour)^2^ + hour + distance

*Note:* Date + hour + time of day + duration + distance + area + minimum temperature + tree density + oak density + oak proportion.

## 3 Results

Transects covered a total of 78.13 km walking in one direction over the course of 33.25 h on campus. We documented 152 individual observations and 197 behavioral observations of fox squirrels. The majority of squirrels were observed in the evening (60%, *n* = 91). However, results of the ANOVA showed no significant difference in observed density of squirrels between early morning (*n* = 58), late morning (*n* = 2), early evening (*n* = 19), and late evening (*n* = 72) categories (*p* = 0.12). Pairwise Tukey's HSD test showed no significant differences between any two time of day categories (0.115 < *p* < 0.972). Results of the Kruskal–Wallis test also found no significant difference in observed squirrel density between time of day categories (*p* = 0.1616). Based on Pearson correlation coefficients (Table 1), both minimum and maximum temperature strongly correlated with hour (PCC = 0.9568 and 0.9605, respectively). Tree and oak density (PCC = 0.8192), as well as distance and area (PCC = 0.6479), was also strongly correlated. Therefore, these pairs of colinear parameters were not used together in modeling.

### 3.1 Tree Species Selection

Selection indices (Table 3) for trees occupied with squirrels indicated preference for most species occupied, but greatest for *Ulmus virginiana* (*D* = 0.92), *Triadica sebifera* (*D* = 0.52), and *Quercus macrocarpa* (*D* = 0.51); *Ulmus parvifolia* (*D* = −0.40) and *Lagerstroemia indica* (*D* = −0.85) were avoided. Selection indices for tree nearest squirrel indicated greatest preference for *Abelia* spp. (*D* = 0.96), *Ulmus virginiana* (*D* = 0.80), *Prosopis glandulosa* (*D* = 0.63), *Liquidambar styraciflua* (*D* = 0.52), and *Triadica sebifera* (*D* = 0.39) and greatest avoidance for *Lagerstroemia indica* (*D* = −0.62), *Ulmus crassifolia* (*D* = −0.45), *Celtis* spp. (*D* = −0.35), and *Ulmus* spp. overall (*D* = −0.30).

**TABLE 3:** Jacobs' (*D*) index of selectivity of tree species that were the nearest tree to a squirrel or were occupied by a squirrel. *NA* values indicate species that were never occupied. Tree species present in the area without any squirrels observed near them are not shown.

Species	Number available	Number with squirrels near	*D* (near)	Number occupied	*D* (occupied)
*Abelia* spp.	1	1	0.96	0	*NA*
*Celtis* spp.	93	1	−0.35	0	*NA*
*Juniperus virginiana*	35	1	0.12	0	*NA*
*Lagerstroemia indica*	1377	8	−0.62	1	−0.85
*Liquidambar styraciflua*	14	1	0.52	0	*NA*
*Pistacia chinensis*	124	2	−0.16	0	*NA*
*Prosopis glandulosa*	10	1	0.63	0	*NA*
*Quercus macrocarpa*	81	4	0.38	2	0.51
*Quercus shumardii*	69	3	0.32	1	0.29
*Quercus* spp.	3515	116	0.29	40	0.26
*Quercus stellata*	433	21	0.39	7	0.35
*Quercus virginiana*	2821	88	0.22	30	0.19
*Taxodium distichum*	125	4	0.18	0	*NA*
*Triadica sebifera*	39	2	0.39	1	0.52
*Ulmus crassifolia*	464	4	−0.45	5	0.15
*Ulmus parvifolia*	285	4	−0.23	1	−0.40
*Ulmus* spp.	804	10	−0.30	7	0.04
*Ulmus virginiana*	5	1	0.80	1	0.92
Unknown sp.	218	1	−0.66	2	0.07

### 3.2 Linear Regression

The most supported and parsimonious models, based on AICc, included distance and hour, with either total tree density or oak density (Table 4). Models including temperature were ranked lower than models including hour, so these were discarded, due to high PCC between the two parameters (> 0.950). Similarly, models including area were ranked lower than models including distance, so these were discarded, due to high PCC.

**TABLE 4:** The two highest‐ranked models, compared against null, global, and single‐parameter models. Models ranked by AICc, including informative covariates predicted to influence Eastern fox squirrel density (*Sciurus niger*).

Parameters	AICc	ΔAICc	AICc Weight	*R* ^2^	Adjusted *R* ^2^
Tree density × hour + distance	843.07	0.00	0.77	0.2744	0.2329
Tree density + hour + distance	845.71	2.64	0.21	0.2243	0.1915
Tree density	851.44	8.37	0.01	0.1106	0.0984
Oak density	852.95	9.88	0.01	0.0925	0.0800
Distance	855.24	12.16	0.00	0.0644	0.0516
Area	856.19	13.12	0.00	0.0524	0.0394
Hour	857.03	13.96	0.00	0.0418	0.0286
Minimum temperature (estimated)	857.60	14.53	0.00	0.0344	0.0212
Null model	858.06	14.98	0.00	*NA*	*NA*
Global model	859.22	16.14	0.00	0.3381	0.2099
Duration	859.47	16.40	0.00	0.0086	−0.0050
Oak proportion	860.11	17.04	0.00	0.0015	−0.0122

**FIGURE 2:**
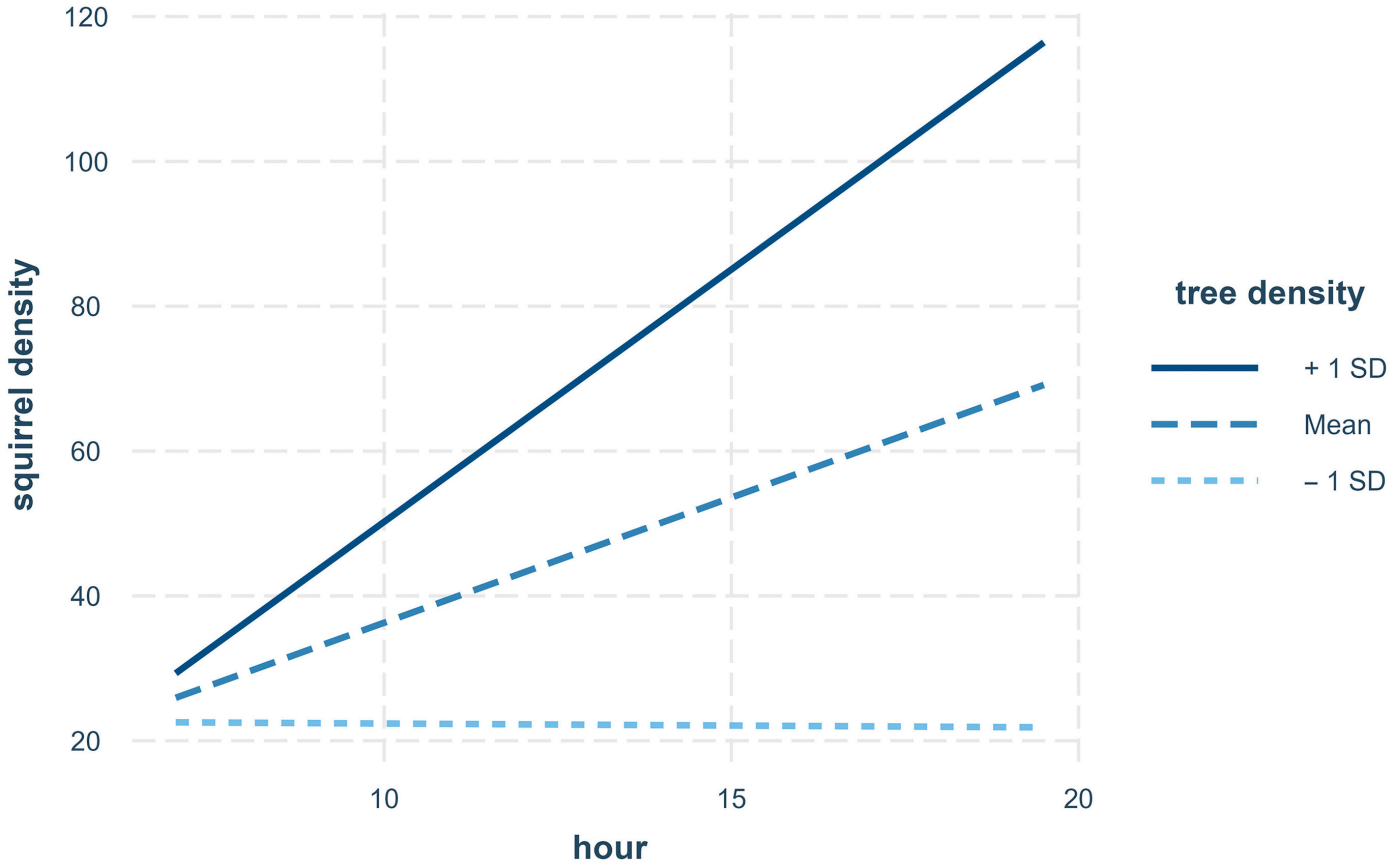
Interaction plot showing the effect of the interaction between tree density and hour on squirrel density in the model squirrel density = tree density × hour + distance.

No models had Δ AICc < 2.00 compared with the top‐ranked model. However, after the second round of model construction, the second‐ranked model was the only model with Δ AICc < 3.00 and AICc weight ≥ 0.10, and cumulative weight of the two highest‐ranked models was 0.49 (Table S1). In the top‐ranked model, tree density and hour were negatively associated with squirrel density, but this was not significant (*p* = 0.3011 and 0.3224, respectively). The interaction between tree density and hour did have a significant (*p* = 0.0313) and positive association with squirrel density (see Figure 2 and Table 5). However, in the second highest‐ranked model, with no interaction term, both tree density (*p* = 0.0094) and hour (*p* = 0.0218) were significantly and positively associated with squirrel density (Table 6). Results for models including oak density instead of tree density were similar, with oak density and hour significantly and positively associated with squirrel density (Table S2). However, the interaction between oak density and hour was not significant (*p* = 0.0696; Table S3). Duration of transect and proportion of oaks were not significant predictors of squirrel density (*p* > 0.05), although the interaction between oak density and oak proportion was significantly and negatively associated with squirrel density (Table S4). Distance of the transect was always negatively associated with squirrel density and was always a significant predictor (*p* < 0.05; Tables 5 and 6).

**TABLE 5:** Coefficients for the top‐ranked model influencing squirrel density (squirrel density = tree density × hour + distance).

	Estimate	SE	*t* value	*p*	
(Intercept)	89.50	43.59	2.053	0.0438	*
Tree density	−0.0131	0.0125	−1.042	0.3011	
Hour	−3.571	3.583	−0.997	0.3224	
Distance	−43.54	18.51	−2.352	0.0215	*
Tree density × hour	0.0022	0.0010	2.197	0.0313	*

*Note:* A single asterisk (*) indicates a *p*‐value ≤ 0.05, while a double asterisk (**) signifies a *p*‐value ≤ 0.01.

**TABLE 6:** Coefficients for the second‐ranked model influencing squirrel density (squirrel density = tree density + hour + distance).

	Estimate	SE	*t* value	*p*	
(Intercept)	15.44	28.37	0.544	0.5880	
Tree density	0.0126	0.0047	2.669	0.0094	**
Hour	3.588	1.529	2.346	0.0218	*
Distance	−52.01	18.58	−2.798	0.0066	**

*Note:* A single asterisk (*) indicates a *p*‐value ≤ 0.05, while a double asterisk (**) signifies a *p*‐value ≤ 0.01.

## 4 Discussion

### 4.1 Main Findings

The five species most preferred as nearest tree to squirrel all had populations below 15 within the sample, which may have inflated selection index values. Specifically, while *Abelia* spp. had the highest selection index (*D* = 0.96), this is based on only one individual tree observed in the study area. Therefore, we made little inference from this value due to the low sample size. Previous research on Texas A&M's campus found high preference for *Q. virginiana* (Live oak) only, with no preference for other *Quercus* species or *Quercus* in general (McCleery et al. 2007). In contrast, selection indices were lower for *Q. virginiana* than *Q. macrocarpa*, *Q. stellata*, *Q. shumardii*, and *Quercus* overall. A possible explanation for this discrepancy is the disproportionately large population of *Q. virginiana* on campus compared to the other *Quercus* species. When a species is highly abundant, such as *Q. virginiana*, individual sightings may be spread over a larger number of trees, which lowers the observed selection index for that species. Conversely, species with smaller populations, such as *Q. macrocarpa* or *U. virginiana*, may have a higher selection index simply because fewer trees are available, so even a few sightings can result in a high index value. This effect, driven by stochasticity and sample size, can result in the appearance of stronger preference for species with fewer available individuals, whereas the use of highly abundant species may be diluted by their large population size.ly 16 of the 71 tree species along the transects had squirrels nearest or on them, and selection indices showed preference for only 12 of those species, excluding unknown species. Most of these species were *Quercus* or *Ulmus*, which supports previous conclusions that fox squirrels in urban areas maintain narrow habitat preferences, particularly relying on seed‐producing trees such as *Quercus* species at Texas A&M University (McCleery et al. 2007) or mixed pine stands in the southeast (Ditgen, Shepherd, and Humphrey 2007).

Although this study was not conducted to investigate temporal patterns of fox squirrel activity, the observed squirrel density increased with hour of the day. While previous research has connected the effect of hour of day to temperature and daylight (Hicks 1949; Jodice and Humphrey 1992; Moore 1957), in this study, models including hour were ranked higher by AICc than models including temperature. However, this is likely because we did not collect consistent temperature data throughout surveys. In contrast to previous studies showing activity peaking around mid‐morning and decreasing when temperatures increased (Hicks 1949; Jodice and Humphrey 1992; Moore 1957), observed densities were higher at later hours, regardless of higher temperatures. Had surveys been conducted in the middle of the day, not just morning and evening, results may have supported bimodal activity patterns found in previous studies (Hicks 1949). Measuring temperature in real time at the exact location of squirrel observations would also have accounted for variations in microclimate, such as cooler temperatures under tree shade, that potentially affected fine‐scale habitat selection. Higher observed densities presumably corresponded to higher detection probability due to higher activity. Activity may have been higher in the evening due to greater shade cover, or it may be an adaptation to avoid people if the college campus is busier earlier in the day. If so, this would be similar to previous findings showing temporal shifts in synanthropes' activity (Ritzel and Gallo 2020).

Linear models also showed that fox squirrel density along transects was positively influenced by tree density and, to a lesser extent, oak density. However, the effect size was only minor, compared with the effects of distance and hour. The proportion of trees that were oaks only had a significant effect in the interaction term with oak density, and this interaction was negative, possibly suggesting that the relative proportion of oak trees was not important if the density of oaks was high enough. Previous research has also identified higher tree density as crucial to fox squirrel persistence in urban landscapes, though with a stronger effect size (McCleery 2010; Moore 1957). The importance of tree density regardless of species does contrast with previous research on the importance of mast‐producing oaks for urban populations (McCleery 2010), but follows research showing little to no preference for certain tree species in rural populations (Perkins and Conner 2004; Brown and Batzli 1984). The interaction of both tree and oak density with hour suggests that greater squirrel densities in areas of greater tree density were best detected later in the day, when there was more activity, and less likely to be detected in the morning. In other words, while squirrel density may have been greater in areas of greater tree density, whether or not this was detected depended on the hour in which a transect was surveyed.

Distance and area of transects had negative effects on observed density, with distance having the largest effect size of any single parameter. Walking pace tended to slow when squirrels were observed, especially when multiple squirrels were observed at once, in order to record data and carefully mark exact location of squirrels. This slower pace meant less distance and area were covered in areas where squirrels were seen. On transects where no squirrels were seen, the observer likely walked faster and was able to cover more ground, resulting in transects that covered greater distance and area within the same amount of time as shorter distance transects where squirrels were seen. It is also possible that because transects were longer and covered more area when pace was quicker, squirrels were more likely to be overlooked, so observer error led to lower reported densities.

### 4.2 Future Research and Implications

Further research should specifically investigate temporal patterns of fox squirrel activity using consistent focal sampling throughout the day. Past studies on fox squirrel activity patterns across different parts of their wide range have shown bimodal patterns in summer and unimodal patterns in winter (Koprowski 1994; Koprowski and Corse 2005; Wassmer and Refinetti 2019) or generally unimodal throughout the year (Wassmer and Refinetti 2016; Sovie et al. 2019). However, individual squirrels' temporal activity patterns may fluctuate from day‐to‐day based on factors such as individual personality, weather, and social interactions (Wassmer and Refinetti 2019). Furthermore, research should compare peak activity times between urban and rural populations in the same area to see if synanthropic squirrels have altered time use within anthropogenic environments. Previous research has found contrasting daily temporal activity patterns between urban and rural populations for species such as Wild Boar (*Sus scrofa*) (Podgórski et al. 2013), Eurasian Blackbird (*Turdus merula*) (Dominoni et al. 2014), and mammalian mesocarnivores (Gálvez et al. 2021). This study was conducted during the summer only, so fox squirrel behavior should be studied during other seasons as well to capture potential seasonal variation in habitat use and the effects of temperature and time of day on activity (Ditgen et al. 2007; Hicks 1949; Wassmer and Refinetti 2019). More information on activity and detection probability of fox squirrels based on both time of day and temperature would improve ideal survey strategies for measuring population density. Quantitative data on area coverage by substrate would allow for similar statistical analysis of the use of different microhabitats, such as concrete sidewalks and grass. Further behavioral data, such as through radio telemetry (McCleery 2010), would provide more information on the amount of time spent on different substrates and how activity on a substrate is affected by the type of behavior. Finally, population surveys in rural areas around College Station would allow for comparison between urban and rural populations. However, line‐transect surveys would likely be inappropriate due to low detection probability in a more forested and brushy area, and camera traps or live‐trapping would be more effective (Greene et al. 2016).

This study found higher fox squirrel densities on an urban college campus than other authors have found in rural environments (Greene et al. 2016). Previous research on urban fox squirrel populations has found similar results (Jodice and Humphrey 1992) and even suggested that urban populations may be a source population for rural and suburban areas (McCleery et al. 2008). Decreased predator presence in urban environments can affect prey species density (Bateman and Fleming 2012; McCleery 2010). However, predators, including Gray Fox and Red‐shouldered Hawk, were observed living on campus while surveys were being conducted. Anthropogenic food sources may also increase urban population densities (Bateman and Fleming 2012; Hansen et al. 2020; McCleery 2010; Shochat, Lerman, and Fernández‐Juricic 2010), but squirrels were never observed eating such food during surveys, and their preference for *Quercus* species suggests they may rely, at least partially, on mast‐producing trees for natural food. A third potential driver is that synanthropes use human‐made structures as shelter (McCleery 2010), but previous research at the College Station campus found that squirrels actually avoided buildings (McCleery et al. 2007). Instead, it is possible that urban tree plantings at Texas A&M University provide suitable habitat by mimicking the natural open‐understory oak savannah that fox squirrels prefer (Jodice and Humphrey 1992; Sexton 1990). Since landscaping at a college campus is purposefully managed and recorded, future investigations of urban wildlife habitat could factor landscaping into the study design and statistical analysis. If groundskeepers specifically manage for wildlife, such methodology could test effectiveness.

The results of this study support the idea that use of urban environments by synanthropic species may be discriminatory and heavily dependent on preservation of specific minimum habitat requirements (Ditgen, Shepherd, and Humphrey 2007; Jodice and Humphrey 1992). However, despite challenges to maintaining populations, urban wildlife hold high value for their potential benefits to human well‐being (Perry et al. 2020). In particular, diversity of nonthreatening species such as fox squirrels in urban areas such as college campuses where people experience high stress can have particularly positive effects on mental health (Cameron et al. 2020; Zhao and Gong 2022). Additionally, urban populations may play a role in conservation of threatened species or subspecies (Jodice and Humphrey 1992). Wildlife managers in urban and suburban areas should consider how meeting particular minimum habitat needs can allow for wildlife persistence in anthropogenic environments.

## Author Contributions

**Daniel A. Whitman:** conceptualization (supporting), data curation (lead), formal analysis (equal), investigation (equal), software (lead), writing – original draft (lead), writing – review and editing (supporting). **Ty J. Werdel:** conceptualization (lead), formal analysis (equal), investigation (equal), project administration (lead), software (supporting), writing – original draft (supporting), writing – review and editing (lead).

## Conflicts of Interest

The authors declare no conflicts of interest.

## Supporting information


Table S1.

## Data Availability

Data and code are openly available on GitHub. https://github.com/Mjavanica/Whitman_Werdel_2024.
