# Supplementary material for: Occurrence of Fox Squirrels Influenced by Fine‐Scale Landscape Characteristics on a College Campus
Source: Ecol Evol. 2024 Oct 27;14(10):e70488. doi: 10.1002/ece3.70488 (PMC11512735; doi:10.1002/ece3.70488)
Supplement: Supplementary file 1 — Table S1. [file ECE3-14-e70488-s001.docx]

Table S1: Models ranked by AICc after the second round of model construction using the most explanatory parameters from the first round of model construction. Five models from the first round of model construction, the highest-ranked by AICc, are included for comparison (squirrel density = tree density*hour; squirrel density = oak density*hour; squirrel density = hour + area; squirrel density = hour + distance; and squirrel density = tree density + hour)

| parameters | AICc | Δ AICc | AICc weight | cumulative weight |
| --- | --- | --- | --- | --- |
| tree density*hour + distance | 843.07 | 0.00 | 0.38 | 0.38 |
| tree density + hour + distance | 845.71 | 2.64 | 0.10 | 0.49 |
| oak density*hour + distance | 846.30 | 3.22 | 0.08 | 0.56 |
| tree density*hour | 846.41 | 3.34 | 0.07 | 0.63 |
| tree density + hour² + distance | 846.60 | 3.53 | 0.07 | 0.70 |
| oak density*oak proportion + hour + distance | 847.27 | 4.20 | 0.05 | 0.75 |
| tree density + hour + distance + oak proportion | 847.47 | 4.40 | 0.04 | 0.79 |
| oak density + hour + distance | 847.49 | 4.41 | 0.04 | 0.83 |
| tree density*hour +duration | 847.84 | 4.77 | 0.04 | 0.87 |
| oak density + hour² + distance | 848.60 | 5.52 | 0.02 | 0.89 |
| oak density*hour | 849.20 | 6.13 | 0.02 | 0.91 |
| tree density*oak proportion + hour + distance | 849.44 | 6.37 | 0.02 | 0.92 |
| oak density + hour + distance + oak proportion | 849.60 | 6.52 | 0.01 | 0.94 |
| hour + area | 850.03 | 6.96 | 0.01 | 0.95 |
| hour + length | 850.58 | 7.51 | 0.01 | 0.96 |
| oak density*oak proportion + length | 851.17 | 8.09 | 0.01 | 0.97 |
| tree density + hour | 851.26 | 8.18 | 0.01 | 0.97 |
| oak density*hour + duration | 851.28 | 8.20 | 0.01 | 0.98 |
| tree density | 851.44 | 8.37 | 0.01 | 0.98 |
| oak density | 852.95 | 9.88 | 0.00 | 0.99 |
| tree density + hour + oak proportion | 852.96 | 9.89 | 0.00 | 0.99 |
| tree density*oak proportion + length | 853.20 | 10.13 | 0.00 | 0.99 |
| oak density*oak proportion + hour | 853.64 | 10.57 | 0.00 | 0.99 |
| oak density + hour + oak proportion | 854.78 | 11.70 | 0.00 | 1.00 |
| tree density + hour² + oak proportion | 855.05 | 11.98 | 0.00 | 1.00 |
| tree density*oak proportion + hour | 855.16 | 12.08 | 0.00 | 1.00 |
| distance | 855.24 | 12.16 | 0.00 | 1.00 |
| area | 856.19 | 13.12 | 0.00 | 1.00 |
| oak density + hour² + oak proportion | 856.82 | 13.75 | 0.00 | 1.00 |
| hour | 857.03 | 13.96 | 0.00 | 1.00 |
| minimum temperature (estimated) | 857.60 | 14.53 | 0.00 | 1.00 |
| global | 857.95 | 14.87 | 0.00 | 1.00 |
| null | 858.06 | 14.98 | 0.00 | 1.00 |
| duration | 859.58 | 16.51 | 0.00 | 1.00 |
| oak proportion | 860.11 | 17.04 | 0.00 | 1.00 |

Table S2: Coefficients for the model squirrel density = oak density + hour + distance

|  | Estimate | SE | t value | *p*-value |  |
| --- | --- | --- | --- | --- | --- |
| (Intercept) | 26.17 | 27.50 | 0.952 | 0.3445 |  |
| oak density | 0.0177 | 0.0077 | 2.301 | 0.0243 | * |
| hour | 3.592 | 1.552 | 2.315 | 0.0235 | * |
| distance | -52.07 | 18.84 | -2.764 | 0.0073 | ** |

Table S3: Coefficients for the model squirrel density = oak density*hour + length

|  | Estimate | SE | t value | *p*-value |  |
| --- | --- | --- | --- | --- | --- |
| (Intercept) | 82.22 | 40.70 | 2.020 | 0.0472 | * |
| oak density | -0.0211 | 0.0223 | -0.942 | 0.3494 |  |
| hour | -1.730 | 3.267 | -0.530 | 0.5981 |  |
| distance | -43.19 | 19.14 | -2.257 | 0.0272 | * |
| oak density*hour | 0.0031 | 0.0017 | 1.843 | 0.0696 | . |

Table S4: Coefficients for the model squirrel density = oak density*oak proportion + hour + distance

|  | Estimate | SE | t value | *p*-value |  |
| --- | --- | --- | --- | --- | --- |
| (Intercept) | -31.80 | 46.90 | -0.678 | 0.4500 |  |
| oak density | 0.0723 | 0.0263 | 2.756 | 0.0075 | ** |
| oak proportion | 83.24 | 68.78 | 1.210 | 0.2304 |  |
| hour | 3.797 | 1.540 | 2.465 | 0.0162 | * |
| distance | -54.37 | 18.55 | -2.932 | 0.0046 | ** |
| oak density*oak proportion | -0.0817 | 0.0384 | -2.127 | 0.0370 | * |
